# Evidence for the gut‐skin axis: Common genetic structures in inflammatory bowel disease and psoriasis

**DOI:** 10.1111/srt.13611

**Published:** 2024-02-13

**Authors:** Jinyan Guo, Qinghua Luo, Chunsheng Li, Hong Liang, Qiurui Cao, Zihao Li, Guanghua Chen, Xuchao Yu

**Affiliations:** ^1^ Department of Anorectal Surgery Jiangmen Wuyi Hospital of Traditional Chinese Medicine Jiangmen China; ^2^ Clinical Medical College Jiangxi University of Chinese Medicine Nanchang China; ^3^ Department of Anorectal Surgery Affiliated Hospital of Jiangxi University of Chinese Medicine Nanchang China

**Keywords:** genetic risk loci, genetic structure, gut‐skin axis, GWAS, psoriasis

## Abstract

**Background:**

Inflammatory bowel disease (IBD) and psoriasis (Ps) are common immune‐mediated diseases that exhibit clinical comorbidity, possibly due to a common genetic structure. However, the exact mechanism remains unknown.

**Methods:**

The study population consisted of IBD and Ps genome‐wide association study (GWAS) data. Genetic correlations were first evaluated. Then, the overall evaluation employed LD score regression (LDSC), while the local assessment utilized heritability estimation from summary statistics (HESS). Causality assessment was conducted through two‐sample Mendelian randomization (2SMR), and genetic overlap analysis utilized the conditional false discovery rate/conjunctional FDR (cond/conjFDR) method. Finally, LDSC applied to specifically expressed genes (LDSC‐SEG) was performed at the tissue level. For IBD and Ps‐specific expressed genes, genetic correlation, causality, shared genetics, and trait‐specific associated tissues were methodically examined.

**Results:**

At the genomic level, both overall and local genetic correlations were found between IBD and Ps. MR analysis indicated a positive causal relationship between Ps and IBD. The conjFDR analysis with a threshold of < 0.01 identified 43 loci shared between IBD and Ps. Subsequent investigations into disease‐associated tissues indicated a close association of IBD and Ps with whole blood, lung, spleen, and EBV‐transformed lymphocytes.

**Conclusion:**

The current research offers a novel perspective on the association between IBD and Ps. It contributes to an enhanced comprehension of the genetic structure and mechanisms of comorbidities in both diseases.

## 1 INTRODUCTION

Inflammatory bowel disease (IBD) is characterized by chronic immune‐mediated inflammation of the gastrointestinal tract, exhibiting either an acute onset or a slow relapse.^1^ Clinical manifestations of IBD include abdominal pain, frequent diarrhea, weight loss, and certain constitutional symptoms.^2^, ^3^, ^4^ In recent decades, Western countries such as Europe and North America have seen a significant increase in the prevalence of IBD.^5^ According to a 2017 statistical report on IBD, a total of 60 000 cases of this disease were reported globally. The prevalence has risen from 79.5 cases per 100 000 individuals in 1999 to 84.3 cases per 100,000 individuals.^6^ In addition, scholars have identified an association between IBD and several other types of immune‐mediated diseases, including psoriasis (Ps). Ps is a chronic, recurrent inflammatory skin disease, mostly manifested as a large number of plaques on the skin and complicated by specific symptoms such as redness, itchiness, and dryness, among others. It is widely regarded as one of the most challenging conditions to treat.^7^

Examining the intricate relationship between Ps and IBD poses challenges when relying solely on conventional epidemiological studies, which are vulnerable to recall bias, misclassification, and residual confounding. One promising strategy for addressing these challenges involves the application of deep learning, a type of machine learning.^8^, ^9^, ^10^, ^11^ Deep learning algorithms have demonstrated the capability to detect subtle changes in skin lesions, enabling rapid and accurate diagnosis of Ps. However, the effectiveness of this approach relies heavily on the availability of high‐quality imaging data, which may not always be readily accessible for research purposes. Another increasingly utilized method is genome‐wide cross‐trait analysis, which leverages publicly available genetic association data.^12^ A comprehensive analysis of genome‐wide association studies (GWAS) on IBD and Ps suggested that Ps and IBD share seven susceptible loci of genome‐wide significance beyond the human leukocyte antigen (HLA) region and possess four shared risk loci (IL23R, IL12B, REL, and TYK2).^13^ CDKAL1 is identified as a shared susceptibility gene for IBD and Ps, contributing to Ps morbidity when its protein expression level is low^14^ and exacerbating IBD.^15^ Currently, only a limited number of genetic risk loci for IBD and Ps have been explored. Our future focus is to comprehensively identify the risk loci associated with the comorbidities of IBD and Ps.

The prior GWAS on Ps and IBD laid the foundation for investigating the genetic mechanisms underlying both diseases.^16^, ^17^, ^18^ In analyses of shared genetic architecture for complex diseases, relying solely on the significant loci identified in GWAS for both diseases, which are prone to false negatives, is deemed unacceptable. This is because their genetic risk architecture is generally based on common genetic variants with small effect sizes.^19^ Fortunately, some novel genetic statisticians can solve this challenge by estimating the correlation of genetic structure between two complex diseases at multiple levels using GWAS summary statistics.^20^, ^21^, ^22^, ^23^ These methods were employed to enhance the understanding of the shared genetic structure and causal relationships between Ps and IBD.

The initial method employed was genetic correlation analysis, utilizing the LD score regression (LDSC) to assess IBD and Ps as a whole^24^ and heritability estimation from summary statistics (HESS) to investigate their local genetic correlations.^25^ Subsequently, Mendelian randomization (MR) was used to infer causalities for both diseases.^26^ Furthermore, the conditional false discovery rate/conjunctional FDR (cond/conjFDR) method was employed to explore potential genetic correlations in the multigene overlap between Ps and IBD. Following this, shared genetic risk loci between the two conditions were identified.^27^ Lastly, tissues linked to Ps and IBD were determined using LDSC applied to specifically expressed genes (LDSC‐SEG). The aforementioned initiatives were expected to shed more light on the common genetic makeup and underlying pathways linking Ps and IBD.

The primary results of this research are as follows:

1. Genetic correlation analysis revealed an overall positive genetic correlation between IBD and Ps, with local genetic correlations identified on chromosomes 1, 5, and 6.
2. Bidirectional MR analysis indicated that IBD causally influences the risk of Ps, but the reverse causal relationship was not observed.
3. Cond/conjFDR analysis identified 43 genetic variants shared between IBD and Ps. Among these, 14 had the same effect direction on both conditions, while 29 had opposite directions.
4. LDSC‐SEG analysis suggested that IBD and Ps share common tissue origins, including whole blood, lung, spleen, and EBV‐transformed lymphocytes.

The paper's organization is structured as follows:

Section 2 explains the data sources and methods employed for genetic analyses.

Section 3 presents the results encompassing genetic correlations, causal relationships, shared genetic variants, and trait‐specific enrichments between IBD and Ps.

Section 4 delves into result comparisons with existing studies, potential biological mechanisms, significance, and limitations.

Finally, in Section 5, the paper concludes by summarizing the results and highlighting areas for potential future research.

## 2 METHODS AND MATERIALS

### 2.1 GWAS data

**FIGURE 1:**
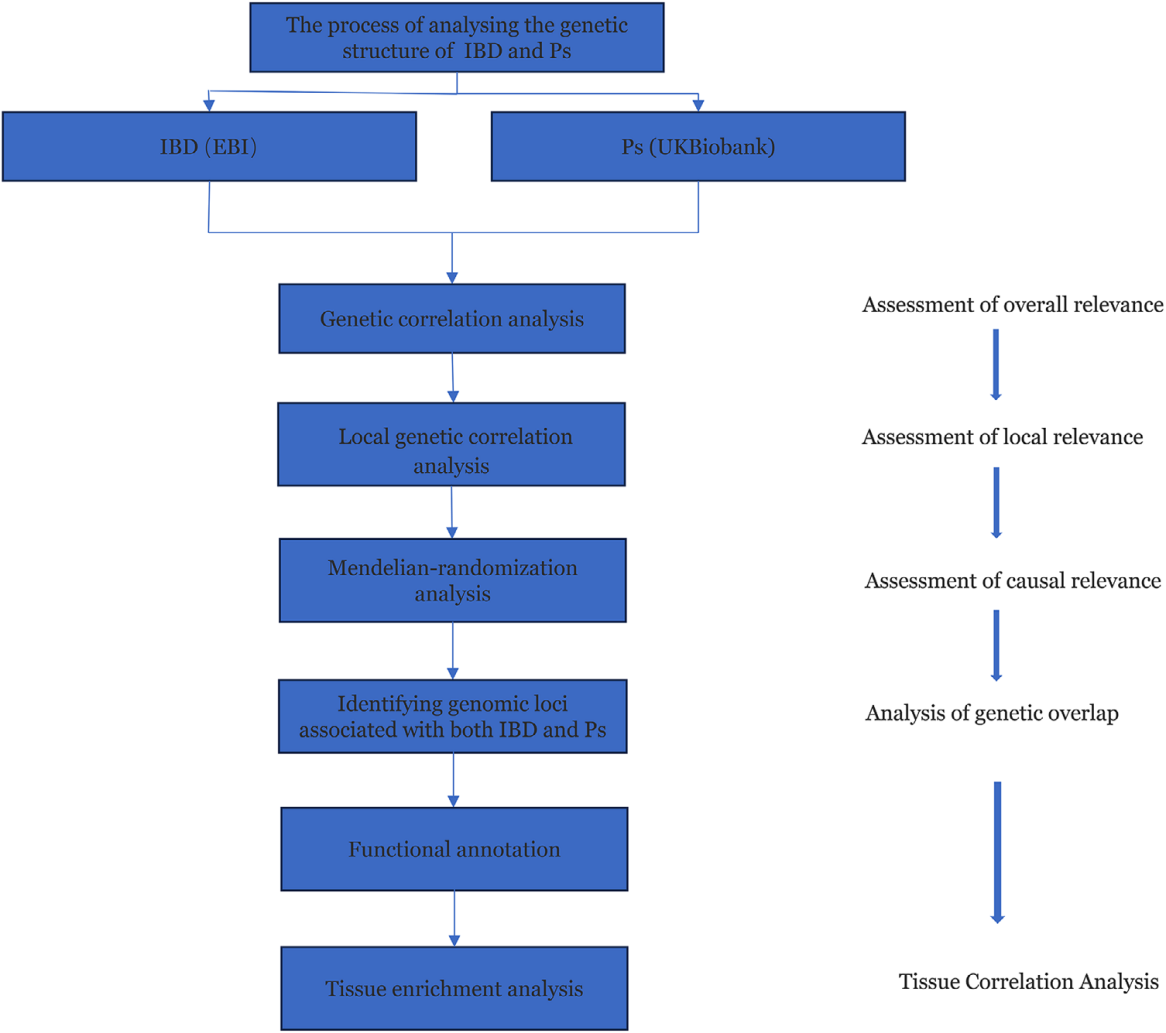
Flowchart of the study. IBD, inflammatory bowel disease; Ps, Psoriasis.

The MRC Integrative Epidemiology Unit at the University of Bristol (IEU) Open GWAS database (https://gwas.mrcieu.ac.uk/datasets/) provided summary statistics for IBD and Ps. Specifically, the summary statistics for IBD (GWAS ID: ebi‐a‐GCST004131) were obtained from a GWAS meta‐analysis, including 25,042 IBD cases and 34 915 controls. The GWAS summary statistics for Ps were sourced from the UK Biobank (UKB) (GWAS ID: ukb‐b‐10537), encompassing 5314 patients and 457 619 controls. The participants from both GWASs were of European ancestry. The flow chart of this study is depicted in Figure 1.

### 2.2 Genetic correlation analysis

LDSC (version 1.0.1) stand out as an efficient method for calculating the genetic correlations between pairs of traits.^28^ The analysis focused on the genetic correlation (rg), which ranged from a minimum of “−1” to a maximum of “+1”, with “‐” and “+” denoting negative and positive correlations, respectively. Following the instructions in the LDSC package, the first step involved using the default parameters of munge_sumstats.p to convert the summary statistics into LDSC format. Subsequently, the ldsc.py tool, with parameters ‐rg, ‐ref‐ld‐chr, and ‐w‐ld‐chr, was utilized to calculate genetic correlations. Pre‐calculated LD score files for the ‐ref‐ld‐chr and ‐w‐ld‐chr flags are available for download at https://alkesgroup.broadinstitute.org/LDSCORE/. The European ancestry information from the 1000 Genomes Project^29^ was selected as the LD reference panel, consistent with the GWAS samples from this study. *p*‐Values of the LDSC were corrected for multiple resting using the Bonferroni method.

### 2.3 Local genetic correlation analysis

The genome components were separated into 1703 segments unrelated to pre‐specified LDs, and the local genetic correlation for each segment was calculated. HESS, a novel computational tool, is commonly used to estimate and express local single‐nucleotide polymorphism (SNP) heritability and genetic correlation. Furthermore, it computes genetic covariance, a metric quantifying the degree of similarity between pairs of traits driven by genetic variation.^25^ The results obtained from HESS underwent correction using the Bonferroni method, and a statistically significant correlation was defined as *p* < 0.05/1703 = 2.94E‐05.

### 2.4 Mendelian‐randomization analysis

**FIGURE 2:**
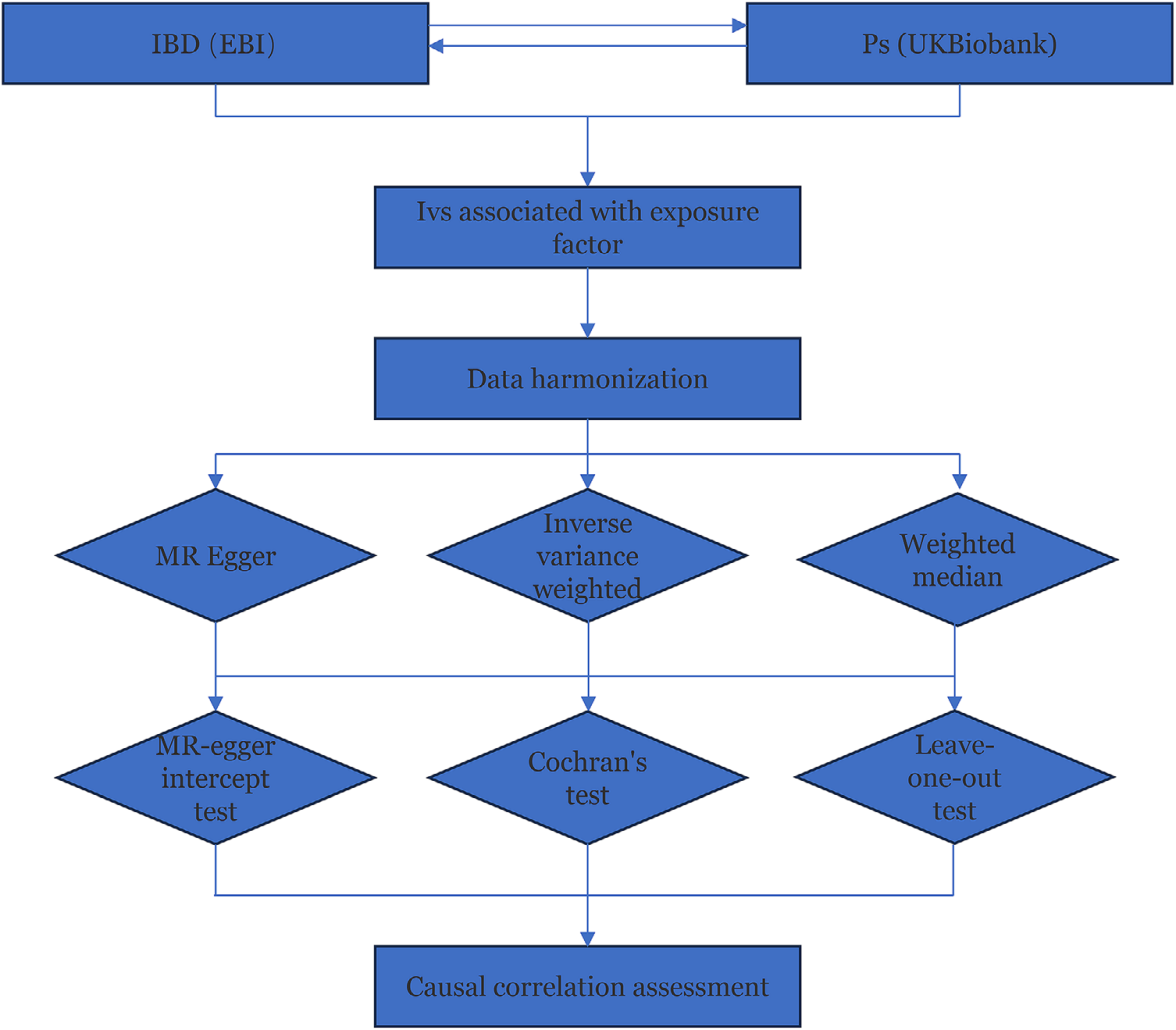
MR Analysis process and acquisition of valid IV steps. IBD, inflammatory bowel disease; IV, instrumental variables; Ps, Psoriasis.

The process of obtaining the effective instrumental variables (IVs) and conducting the Mendelian Randomization (MR) analysis is shown in Figure 2. To determine SNPs linked to exposure and meet the correlation assumption of Mendelian Randomization, the *p*‐value was set to be less than 5 × 10^−8^. Secondly, to eliminate the influence of linkage disequilibrium (LD), kilobase (kb) was set to 10 000, and *r* ^2^ was set to 0.001.^30^

MR controls the quality of analyses in terms of multiplicity, heterogeneity, and sensitivity. In this study, multiplicity was assessed by the Mendelian randomization‐Egger (MR‐Egger) regression^31^ and the Mendelian Randomization Pleiotropy RESidual Sum and Outlier (MR‐PRESSO) test.^32^ The latter detected anomalous SNPs, leading to their exclusion from the analysis. Heterogeneity was assessed using the Inverse variance weighted (IVW) and MR Egger methods. Cochran's Q statistics and corresponding *p*‐values were determined, with the *p*‐value serving as a criterion for assessing the presence or absence of heterogeneity (*p* < 0.05 for none). Leave‐one‐out (LOO) analysis was conducted by systematically excluding each SNP one at a time, recalculating the combined effect of the remaining SNPs, and assessing whether the exclusion of a specific SNP had a detrimental impact on the overall causal association.^33^ In addition, the *F*‐statistic was calculated to assess the strength of IVs using the following formula: *F* = β2/SE2, where β is the effect estimate of the risk allele, and SE is the standard error of the effect estimate.^34^ An *F*‐statistic above 10 indicated that the IVs were eligible and that there was no influence of weak IV bias.^35^

MR analyses were performed using the TwoSampleMR R package, employing three methods under different assumptions (IVW, MR Egger, and Weighted median [WM]). The IVW method is the most critical approach for assessing the causal associations by calculating the Wald estimates for each SNP.^36^ In contrast, the MR‐Egger approach evaluates the pleiotropy of IVs and performs weighted regressions that consider nodal increment, with the slopes providing estimates of the causal effects.^37^ WM is effective in mitigating large differences in estimation accuracy, utilizing the inverse variance weights of each genetic variant.^38^ The MR analyses performed in this study were bidirectional.

### 2.5 Identifying genomic loci associated with both IBD and Ps

Genetic risk loci shared by Ps and IBD were screened using cond/conjFDR analyses based on the empirical Bayesian approach.^39^ Initially, a conditional quantile‐quantile plot (conditional Q‐Q plot) was used to show how SNPs overlapped across various traits. The principle behind this analysis was to set SNPs with secondary traits as the reference and then evaluate all SNPs with primary traits. In the second step, the condFDR statistical framework was used to reorder the test statistics of primary traits (such as IBD) based on how strongly they were associated with secondary traits (such as Ps). This approach improved the ability to identify SNPs.^27^ Next, to obtain the reverse condFDR, the primary and secondary traits were reversed. The significance threshold for the primary and secondary significant loci was set at “condFDR ≤ 0.01.” Finally, the maximum value of condFDR was set as the value of conjFDR to ensure that the FDR of the two traits was accurate. Specifically, SNPs around the extended region of the major histocompatibility complex (MHC) were directly removed, reducing bias in the FDR estimates.^40^ Detailed analysis procedures for conjFDR are available at https://github.com/precimed/pleiofdr.

### 2.6 Functional annotation

This study mapped and functionally annotated the novel, shared, and specific loci identified in this screening using the FUMA platform (https://fuma.ctglab.nl/).^41^ The specific process involved uploading the loci‐related SNP information to the SNP2Gene module of FUMA and then retrieving the mapped genes. Enrichr, an online platform, was utilized to analyze these mapped genes for functional enrichment. (https://amp.pharm.mssm.edu/Enrichr/).

### 2.7 Tissue enrichment analysis

To determine tissues linked to IBD and Ps, LDSC‐SEG analysis using tissue gene expression data was performed.^28^ This involved analyzing tissue gene expression data alongside GWAS summary statistics.^42^ *t*‐Statistics for each gene expressed in a particular tissue were determined using LDSC‐SEG. All genes were ranked according to the *t*‐statistics score, and the top 10% of genes exhibiting the most elevated *t*‐statistics were defined as the gene set corresponding to the focal tissue. Based on intensity, this gene set was classified as either dominant or recessively expressed genes. Next, to construct a functional annotation for each gene in each gene set, 100 kb windows were set on the anterior and posterior sides of the transcribed regions of each gene. Finally, GWAS was applied to assess the role played by key genome annotations in trait heritability. Genotype‐Tissue Expression (GTEx) project served as the genome annotation during the analysis.^42^ The comprehensive analysis procedure for LDSC‐SEG is available at https://github.com/bulik/ldsc/wiki/Cell‐type‐specific‐analyses.

## 3 RESULTS

### 3.1 Genetic correlation

In the LDSC analysis, 2380 SNPs were significantly associated with IBD, and 490 were associated with Ps. Regardless of the intercept, the heritability of Ps was 0.66%, and that of IBD was 31.76%. Notably, a positive association was found between IBD and Ps (*rg* = 0.2347, *p* = 0.0024).

**FIGURE 3:**
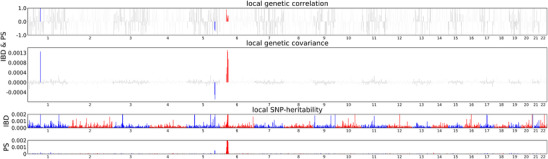
HESS analysis of IBD and Ps. The top and middle sections display local genetic correlations and covariances between IBD and Ps, respectively. Genetic correlation, denoted as rg, is the statistical measure of the degree to which two traits share common genetic influence. The coloured bars indicate loci with significant local genetic correlations and covariances. The bottom portion represents the local SNP heritability of each trait (IBD and Ps), with coloured bars denoting loci with significant local SNP heritability. Heritability refers to the proportion of the total variance in a trait that can be attributed to genetic variation. HESS, heritability estimation from summary statistics; IBD, inflammatory bowel disease; Ps, psoriasis; SNP, single‐nucleotide polymorphism.

According to the analysis of local genetic correlation, there was a local genetic overlap between IBD and Ps in the three chromosomes 1, 5, and 6 (Figure 3), indicating a local correlation between them. The results are shown in Table S1.

### 3.2 Mendelian randomization

**FIGURE 4:**
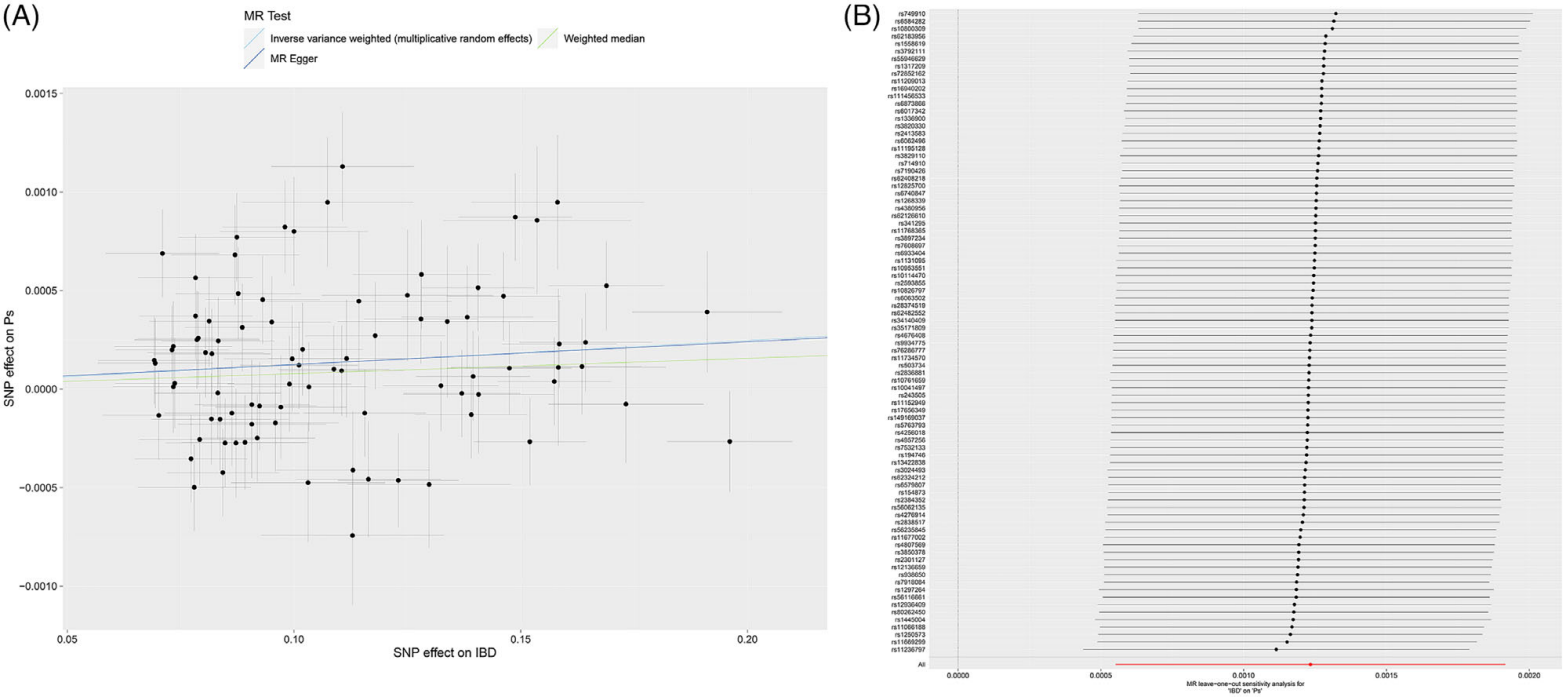
Results of MR analyses. (A) Scatter plot illustrating the causal effect of IBD on Ps. Each point represents a genetic variant, and the slope of each line corresponds to the effect estimates obtained by different MR methods. (B) Forest plot displaying the results of the leave‐one‐out analysis. Each genetic variant was systematically removed to assess whether the overall MR estimates were influenced by a single genetic variant. IBD, inflammatory bowel disease; MR, Mendelian randomization; Ps, psoriasis.

Using bidirectional MR analysis, the causalities of Ps and IBD were determined. To determine the causal link between IBD and Ps, 86 SNPs were examined. The IVW results clearly demonstrated that IBD had a positive causal effect on Ps risk (*p* = 3.92E‐04) (Figure 4A, Table S2). LOO analyses revealed no potentially influential SNPs causing causal relationships, indicating the validity of the findings of this study (Figure 4B). Additionally, MR‐PRESSO detected no horizontal pleiotropy (*p*‐value of 0.95, > 0.05) (Table S3). Heterogeneity was observed in the study (Table S4). Upon performing reverse MR analyses, no noteworthy indication of a causal relationship between Ps and IBD was detected (Table S2). The IV‐related data involved in this study are shown in Tables S5–6. The *F*‐statistics for all IVs were > 10, suggesting no evidence of weak instrument bias (Tables S5–6).

### 3.3 ConjFDR analysis identifies shared genomic loci between IBD and Ps

**FIGURE 5:**
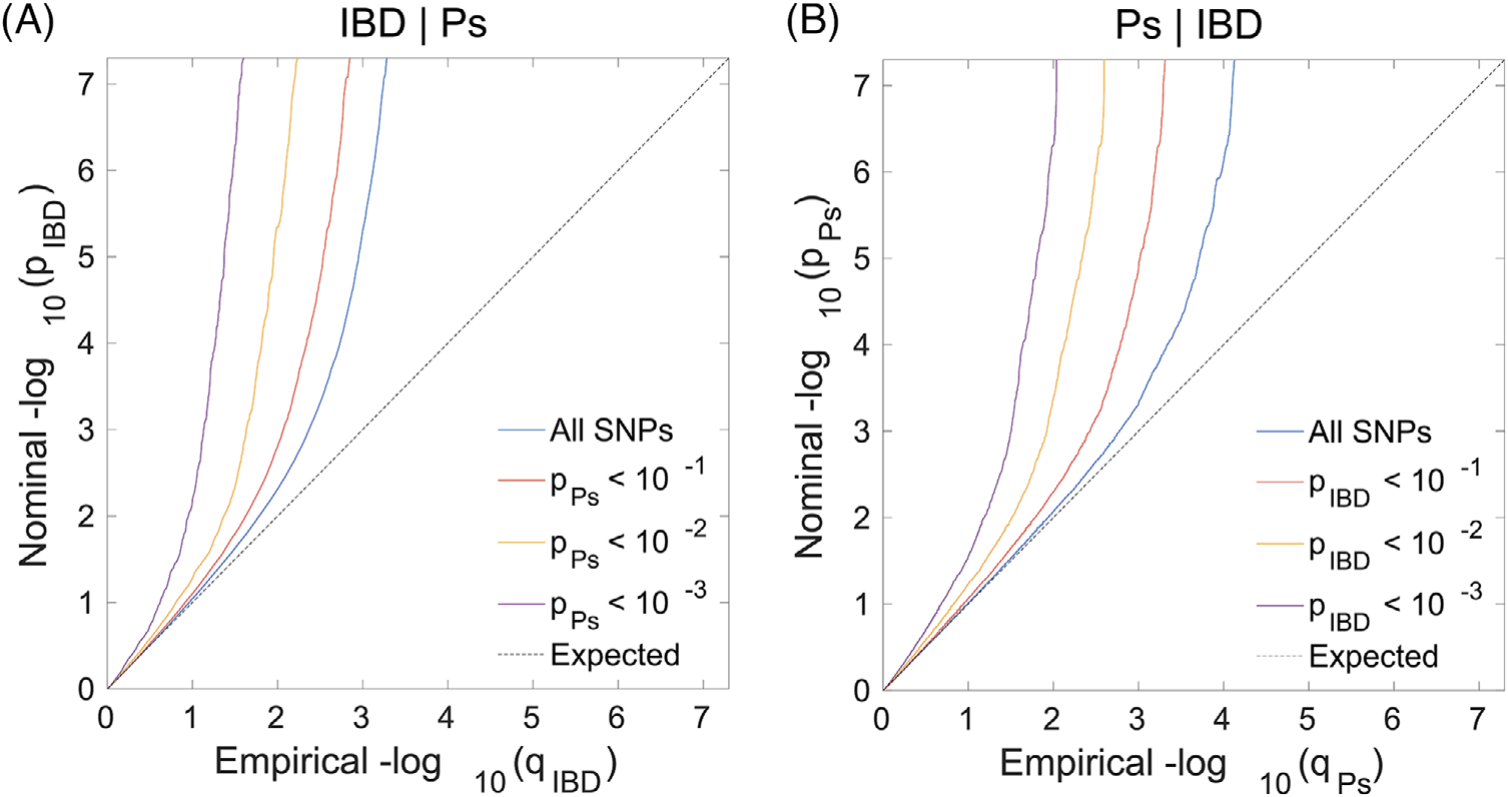
Conditional quantile‐quantile plot. The dashed line indicates the expected line under the null hypothesis, and the deflection to the left indicates the degree of pleiotropic enrichment. IBD, inflammatory bowel disease; Ps, Psoriasis.

The conditional Q–Q plot for IBD and Ps visually demonstrated that as the association significance of one disease increases, the degree to which the other disease is deflected to the left from the expected zero line increases dramatically, suggesting a close correlation between the phenotypes of IBD and Ps (Figure 5). The multigene overlap between IBD and Ps further indicated a possible genetic enrichment and shared genes between the two conditions.

**FIGURE 6:**
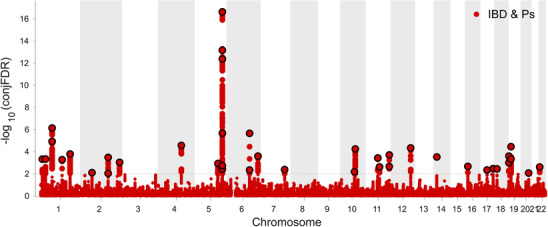
ConjFDR Manhattan plot. The shared risk loci between IBD and Ps were marked. The statistically significant causality is defined to be conjFDR  < 0.01. IBD, inflammatory bowel disease; Ps, Psoriasis.

ConjFDR analyses treated all genes between the two traits in an overlapping manner, enhancing qualitative confidence. The identified loci included both loci specific to a single trait and loci common to both. When the ConjFDR was < 0.01, IBD GWAS shared 43 loci with Ps GWAS (Figure 6, Table 1). Notably, it was observed that 14 lead SNPs had the same effect direction on IBD and Ps, while 29 had the opposite direction (Table 1).

**TABLE 1:** The 43 LD‐independent loci jointly associated with IBD and Ps identified by conjFDR analyses.

SNP	A1	A2	Gene	function	CADD	zscore_IBD	zscore_Ps	conjfdr_IBD_Ps	pval_IBD	pval_Ps
rs6125877	A	G	TRERNA1	downstream	0.013	16.56856	4.617776	0.000243	1.18E‐61	3.88E‐06
rs5754100	T	G	UBE2L3	ncRNA_intronic	0.46	16.475231	4.617776	0.000243	5.53E‐61	3.88E‐06
rs118115488	G	A	PDE4A	ncRNA_intronic	3.861	16.481794	4.581618	0.000284	4.96E‐61	4.61E‐06
rs11672021	T	G	PDE4A	ncRNA_intronic	7.249	16.448919	4.581618	0.000284	8.54E‐61	4.61E‐06
rs35138525	G	T	RP11‐770G2.5	intergenic	1.889	4.241125	−4.458299	0.000471	2.22E‐05	8.26E‐06
rs71639217	A	G	RP11‐431K24.1	intergenic	0.079	−4.216824	4.452106	0.000483	2.48E‐05	8.50E‐06
rs2074452	C	A	SBNO2	ncRNA_intronic	7.996	16.645986	4.42622	0.000535	3.24E‐62	9.59E‐06
rs35665378	G	T	POLR2E	ncRNA_intronic	1.647	16.987018	4.201433	0.00129	1.02E‐64	2.65E‐05
rs2847266	T	C	RP11‐973H7.1	ncRNA_intronic	3.007	16.918868	4.201433	0.00129	3.27E‐64	2.65E‐05
rs111585196	T	C	CYTH1	ncRNA_intronic	7.415	16.879043	4.182069	0.00138	6.42E‐64	2.89E‐05
rs2021511	T	C	RMI2	upstream	0.508	16.795431	4.155728	0.00153	2.64E‐63	3.24E‐05
rs696	A	G	NFKBIA	upstream	0.344	16.842921	4.14756	0.00158	1.18E‐63	3.36E‐05
rs8072566	T	C	STAT3	ncRNA_exonic	1.649	16.817379	4.117458	0.00176	1.82E‐63	3.83E‐05
rs61907765	A	G	ETS1	upstream	3.397	16.691497	4.110494	0.0018	1.51E‐62	3.95E‐05
rs10774624	T	C	RP3‐473L9.4	upstream	5.477	16.711123	4.090739	0.00195	1.09E‐62	4.30E‐05
rs10059288	A	G	AC008697.1	intergenic	1.544	3.781152	−4.14756	0.00232	0.000156	3.36E‐05
rs4921230	A	G	LINC01845	intergenic	0.619	3.71533	−4.14756	0.00294	0.000203	3.36E‐05
rs11221249	G	A	LINC02098	upstream	3.848	−10.369931	−3.926914	0.00354	3.40E‐25	8.60E‐05
rs2052352	T	C	CAMK2G	intergenic	0.551	−4.908544	3.877204	0.00422	9.18E‐07	0.000106
rs12724239	A	G	DENND1B	upstream	0.101	−4.753317	3.851966	0.00462	2.00E‐06	0.000117
rs78703675	A	G	RORC	intergenic	0.739	−4.801241	3.851966	0.00462	1.58E‐06	0.000117
rs10193928	C	T	LINC01185	ncRNA_intronic	0.15	−4.743749	3.82901	0.005	2.10E‐06	0.000129
rs17716942	A	G	KCNH7	intergenic	0.481	−4.77829	3.82901	0.005	1.77E‐06	0.000129
rs72673802	G	A	SLC35D1	intergenic	1.903	−4.803602	3.82901	0.005	1.56E‐06	0.000129
rs11236797	C	T	RP11‐672A2.7	upstream	0.818	3.563157	−4.211751	0.00502	0.000366	2.53E‐05
rs4728142	G	A	IRF5	intergenic	3.007	3.522565	−4.132033	0.00576	0.000427	3.60E‐05
rs1004819	G	A	IL23R	intergenic	1.536	−4.754852	3.788472	0.00577	1.99E‐06	0.000152
rs367254	C	T	TRAF3IP2	intergenic	0.038	3.519888	−4.155728	0.00582	0.000432	3.24E‐05
rs17674015	C	A	CTB‐1I21.1	intergenic	2.084	3.503015	−4.132033	0.00616	0.00046	3.60E‐05
rs76198205	C	T	SLC16A10	intergenic	0.533	3.499623	−4.172955	0.00623	0.000466	3.01E‐05
rs10056700	A	G	RAD50	intergenic	0.704	3.469422	−4.139668	0.00688	0.000522	3.48E‐05
rs9989746	A	G	SP140	intergenic	6.206	−6.033795	3.737534	0.0069	1.60E‐09	0.000186
rs6861973	T	C	RP11‐431K24.1	intergenic	0.063	3.458696	−4.155728	0.00714	0.000543	3.24E‐05
rs13169581	A	G	AC008697.1	intergenic	0.369	3.455223	−4.124634	0.00723	0.00055	3.71E‐05
rs6887065	C	A	AC008697.1	intergenic	6.846	3.451672	−4.155728	0.00731	0.000557	3.24E‐05
rs34470843	G	A	CTB‐1I21.1	intergenic	1.623	3.438201	−4.172955	0.00764	0.000586	3.01E‐05
rs212396	C	T	RP1‐111C20.4	intergenic	1.465	3.435589	−4.124634	0.00771	0.000591	3.71E‐05
rs4921488	A	G	AC008697.1	intergenic	1.711	3.419159	−4.14756	0.0081	0.000628	3.36E‐05
rs2063113	T	G	RP11‐84D1.2	intergenic	1.251	−5.201167	3.669873	0.00869	1.98E‐07	0.000243
rs942793	C	T	ZMIZ1	intergenic	7.825	3.396983	−4.182069	0.00875	0.000681	2.89E‐05
rs7718670	T	C	RNF145	intergenic	0.883	3.381029	−4.155728	0.00922	0.000722	3.24E‐05
rs2633310	A	G	CAMK2G	intergenic	0.578	3.368994	−4.182069	0.00957	0.000754	2.89E‐05
rs13132933	C	T	RN7SL335P	intergenic	4.037	3.361595	−4.139668	0.00979	0.000775	3.48E‐05

*Note*: The table presents reference allele (A1), alternative allele (A2), allele frequency of reference allele, and gene and its functional category. CADD score was used for predicting the deleteriousness of variants. The conjunctional FDR (conjFDR) columns report the maximum condFDR value, from each pair of condFDR analyses. *Z*‐scores and *p*‐values from the original summary statistics on IBD and Ps were also shown.

Abbreviations: IBD, inflammatory bowel disease; LD, linkage disequilibrium; Ps, Psoriasis.

### 3.4 Functional annotations

Using a concFDR of less than 0.01 yielded 1885 candidate SNPs for loci shared by Ps and IBDs. These primarily comprised ncRNA_intronic (*n* = 228.12.20%), intronic (*n* = 813, 43.6%), and intergenic (*n* = 678, 36.4%) sequences. A total of 121 genes were mapped (Table S7). Among the shared loci between IBD and Ps, 26 of the 43 most important lead SNPs were located in intergenic, 8 in ncRNA_intronic, 7 in upstream, 1 in ncRNA_exonic, and 1 in downstream (Table 1). Among these 43 prominent lead SNPs, none of the corresponding CADD values exceeded the threshold score of 12.37, suggesting no deletion.^29^

**FIGURE 7:**
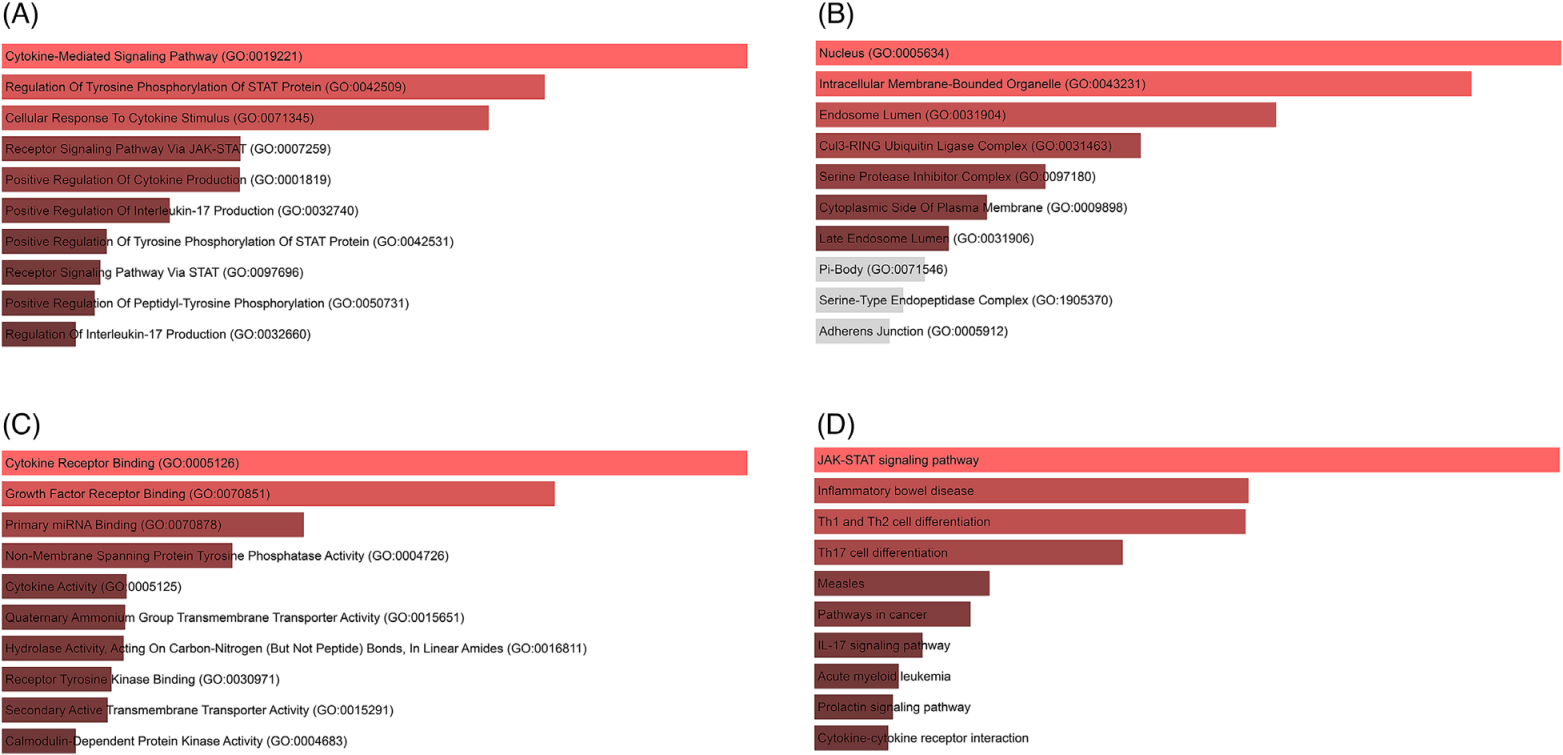
The Results of Enrichment analysis. (A) GO enrichment analysis at biological process. (B) GO enrichment analysis at molecular function. (C) GO enrichment analysis at cell composition. (D) KEGG analysis. GO; Gene Ontology; KEGG, Kyoto Encyclopedia of Genes and Genomes.

Gene Ontology (GO) annotation and Kyoto Encyclopedia of Genes and Genomes (KEGG) pathway enrichment analyses were executed on 121 mapped genes. The results showed that in terms of biological processes, the top 3 genes mainly enriched in Cytokine‐Mediated Signaling Pathway, Regulation Of Tyrosine Phosphorylation Of STAT Protein, and Cellular Response To Cytokine Stimulus (Figure 7A). Regarding cellular composition, the Nucleus, Intracellular Membrane‐Bounded Organelle, and Endosome Lumen were the main directions of gene enrichment (Figure 7B). Finally, in terms of molecular function, genes were mainly enriched in Cytokine Receptor Binding, Growth Factor Receptor Binding, and Primary miRNA Binding (Figure 7C). On the other hand, the significant pathways identified through KEGG analysis included the JAK‐STAT signaling pathway, IBD, and Th1 and Th2 cell differentiation (Figure 7D).

### 3.5 Trait‐related tissue

**FIGURE 8:**
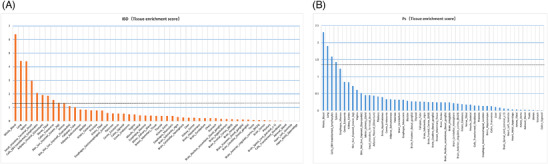
Tissues enrichment results of IBD (A) and Ps (B) using gene expression data of 53 tissues from GTEx. IBD, inflammatory bowel disease; Ps, Psoriasis.

Ultimately, additional LDSC‐SEG analyses using GTEx were carried out to identify tissues linked to the diseases. When *p* < 0.05, tissues associated with IBD included whole blood, lung, spleen, small intestine terminal ileum, EBV‐transformed lymphocytes, adipose visceral (Omentum), nerve tibial, transverse colon, sun‐exposed skin (lower leg), and uterus (Figure 8A). Tissues significantly associated with Ps consisted of whole blood, lung, EBV‐transformed lymphocytes, and spleen (Figure 8B). Notably, whole blood, lung, spleen, and EBV‐transformed lymphocytes were all linked to both IBD and Ps, suggesting a common tissue origin for the two traits, the specific analyses of which are shown in Table S8–9.

## 4 DISCUSSION

In this study, both LDSC and HESS analyses were conducted at the genome‐wide level, and it was concluded that there is an overall and local genetic correlation between IBD and Ps. The investigation of novel genetic variants linked to the two shapes of Ps and IBD utilized summarized data from a large‐scale GWAS and the conjFDR method, revealing consistent genetic loci for both conditions. The histology of Ps and IBD was corroborated by LDSC‐SEG analyses at the tissue and immune cell levels, identifying four common tissue sources: whole blood, lung, spleen, and EBV‐transformed lymphocytes. The findings of this study deepen the comprehension of the genetic contribution of IBD and Ps, reveal the overlapping etiology and course of the two conditions, and offer insights into the possible regulatory roles of the common genetic factors, meriting further investigation.

Consistent with previous studies, this may be explained by the similar pathogenesis of these diseases.^43^, ^44^ Firstly, they share common genetic risk loci.^9^, ^10^, ^11^ Secondly, IBD and Ps are interconnected through the “gut‐skin axis,” a recent focus in biological studies that highlights the direct correlation between inflammation and intestinal dysfunction and the skin. This mirrors the possible genetic overlap between Ps and IBD that was observed in this study. Changes in the gut microbiota and increased intestinal permeability can dysregulate immune homeostasis, which may, in turn, lead to inflammatory skin lesions.^45^ The gut microbiota also affects signaling pathways for epidermal differentiation, thereby altering skin homeostasis.^46^ Thirdly, the immune mechanism linking IBD and Ps may involve dysbiosis, which is a possible common pathogenic pathway that leads to enhanced Th17‐driven immune responses in genetically susceptible hosts.^47^ In addition, IL‐23 promotes the proliferation and survival of Th17 cells while inducing the release of the corresponding cytokines, thus acting as a key cytokine and regulator in both immune disorders.^48^, ^49^ GO enrichment analysis shows that the “Cytokine‐Mediated Signaling Pathway” is the most biologically significant pathway of the two, consistent with the abovementioned performance.

The results of the KEGG pathway enrichment analysis are noteworthy, with the Janus kinase (JAK)‐signal transducer and activator of transcription (STAT) pathway (JAK/STAT pathway) emerging as the most prominent. The typical sequence of events involves the initial interaction of the cytokine with the corresponding cell surface receptor, leading to a conformational change in its intracellular structural domains. This alteration results in the phosphorylation of JAK in the cell. Phosphorylated JAK leads to the activation, phosphorylation, and dimerization of STAT. Subsequently, these STAT dimers migrate to the nucleus as homo‐/heterodimers and bind to specific DNA sites. This induces gene transcription and the production of various cytokines implicated in multiple inflammatory immune responses or functions.^50^ The JAK/STAT pathway has been involved in the pathogenesis of IBD and Ps. First‐generation JAK inhibitors (tofacitinib)^51^ and second‐generation JAK inhibitors (solcitinib) have been developed and are being considered for the treatment of IBD and Ps.^52^ In addition, Th1 and Th2 cell differentiation plays a crucial role in the pathogenesis of IBD and Ps.^53^, ^54^

In two phenotypic conjFDR analyses, 43 loci were identified, with the two most significant SNPs located in the TRERNA1 and UBE2L3 genes. In two GWAS, it was discovered that UBE2L3 may be a novel IBD risk gene that is crucial to the pathogenesis of intestinal disorders.^55^, ^56^ Additionally, a study examining gene expression profiling of inflammatory pathway‐related mediators in intestinal tissues of UC patients revealed a considerably elevated mRNA gene expression level of UBE2L3 relative to the control group (*p* < 0.05).^57^ UBE2L3 is an indirect target of pro‐inflammatory cytokines (IL‐1β) that affects epidermal lesions in patients with Ps.^58^ Translation‐regulated RNA 1 (TRERNA1) is a lncRNA that enhances the transcriptional activity of the EMT transcription factor Snail, with current studies primarily associating it with cancer.^59^ Future immunological studies should be conducted to explore its potential link with IBD and Ps.

LDSC‐SEG also revealed important findings. The examination of gene levels, circulating cytokines, and microRNAs in whole blood shows the pathology of IBD.^60^, ^61^, ^62^ Notably, both the lungs and the gut belong to the common mucosal immune system (CMIS), and there is a lung‐gut crosstalk between them. Patients with IBD may suffer from a spectrum of respiratory diseases, including asthma, allergies, chronic obstructive pulmonary disease (COPD), and respiratory infections.^63^ The spleen, being the largest lymphoid organ, regulates the systemic immune system. Previous studies have mentioned that the spleen can be used to target H_2_S donor‐delivering liposomes for effective immunotherapy of IBD.^64^ EBV‐transformed lymphocytes can be involved in the pathological process of IBD by influencing the secretion of IL6 and IL10.^65^, ^66^ Additionally, the correlation between inflammasomes and microRNAs in whole blood with the pathogenesis of Ps has been established.^67^, ^68^ Patients with Ps show an increased prevalence of interstitial lung disease (ILD) and may exhibit altered splenic conditions in certain cases.^69^, ^70^ In summary, the findings of this research align with prior evidence.

The present study adopted the conjFDR method to assess the directional association of each overlapping genetic variant. This study, independent of genetic correlation, is superior to traditional genetic correlation analyses, but it has some limitations. Firstly, preventing lead SNPs from having LD with other causative SNPs is challenging. While LDSC, HESS, MR, and conjFDR were utilized to mitigate the interference caused by sample overlap, the potential inflation of cross‐trait enrichment results by overlapping participants still persists. In addition, variations in neuropathological processes due to intrinsic interactions between genes and the environment, epistatic effects, and other pathological pathways contribute to the pathological effects observed in IBD and Ps. Finally, since this study relies on a computer simulation approach using publicly accessible GWAS data, a more comprehensive evaluation of the shared genetic architecture between IBD and Ps necessitates functional characterization using independent experimental data and validation in a separate cohort.

## 5 CONCLUSION

In summary, the outcomes of this research demonstrate a genetic correlation between IBD and Ps, offering fresh insights into the genetic relevance of both diseases. This evidence suggests a potential genetic foundation for the clinical link between IBD and Ps. Further investigations using larger and more diverse GWAS samples are anticipated to elucidate the underlying genetic basis, paving the way for exploring treatment options for the comorbidities of IBD and Ps.

## CONFLICT OF INTEREST STATEMENT

The authors declare that the research was conducted in the absence of any commercial or financial relationships that could be construe as a potential conflict of interest.

## ETHICS STATEMENT

Not applicable.

## Supporting information

Supporting Information

## Data Availability

All the GWAS data and statistical software used in this study were publicly available (which can be accessed through the following URLs), and all the generated results in this study were provided in the main text and supplemental data. IEU database: https://gwas.mrcieu.ac.uk, LDSC: https://github.com/bulik/ldsc, LDSC‐SEG: https://github.com/bulik/ldsc/wiki/Cell‐type‐specific‐analyses, TwosampleMR: https://mrcieu.github.io/TwoSampleMR/, conjFDR: https://github.com/precimed/pleiofdr, FUMA: https://fuma.ctglab.nl, Enrichr:https://amp.pharm.mssm.edu/Enrichr/.
